# Role for plant foods in kidney health among middle-aged individuals environmentally exposed to cadmium and lead

**DOI:** 10.1371/journal.pone.0350506

**Published:** 2026-05-29

**Authors:** Donrawee Waeyeng, Supabhorn Yimthiang, Phisit Pouyfung, Soisungwan Satarug, Tanaporn Khamphaya

**Affiliations:** 1 Environmental Health and Technology Program, School of Public Health, Walailak University, Nakhon Si Thammarat, Thailand; 2 Excellence Center for Public Health Research: EC for PHR, Walailak University, Nakhon Si Thammarat, Thailand; 3 Health, Environment and Safety Program, School of Public Health, Walailak University, Nakhon Si Thammarat, Thailand; 4 Research Center for Intelligent Technology and Integration, Walailak University, Nakhon Si Thammarat, Thailand; 5 Occupational Health and Safety Program, School of Public Health, Walailak University, Nakhon Si Thammarat, Thailand; 6 Kidney Disease Research Collaborative, Centre for Health Services Research, The University of Queensland Translational Research Institute, Brisbane, Australia; King Faisal Specialist Hospital and Research Center, SAUDI ARABIA

## Abstract

Plant food consumption has been associated with potential kidney health benefits; however, in polluted environments, such benefits may be diminished by exposure to toxic metals, notably, cadmium (Cd) and lead (Pb). Herein, plant food intake levels were investigated in relation to a simultaneous Cd/Pb exposure and kidney injury, reflected by urinary N-acetyl-β-D-glucosaminidase (uNAG). A cross-sectional study was conducted from October to December 2024 in southern Thailand, where ninety-six residents, aged ≥50 years were recruited during routine chronic disease screening. Consumption of plant foods was assessed using Plant Food Scores (PFS). Blood Cd and blood Pb levels were indicators of exposure to the metals. Estimated glomerular filtration rate (eGFR), blood urea nitrogen (BUN), urine creatinine, and uNAG were indicative of kidney function. ANOVA was used to compare PFS and kidney function indicators in Cd/Pb exposure groups, while associations of PFS, blood Cd, blood Pb and kidney function indicators were evaluated by multivariable regression. BUN levels rose in high Cd/Pb exposure group (12.64 to 15.79 mg/dL; p = 0.016), whereas eGFR and uNAG exhibited non-significant trends suggestive of renal stress. Total PFS was inversely associated with uNAG (β = −0.297; p = 0.010), and higher consumption of nuts and seeds was associated with a lower likelihood of elevated uNAG levels (OR = 0.185; 95% CI, 0.040–0.860; p = 0.032). Thus, consumption of plant foods, particularly nuts and seeds, was associated with lower levels of kidney tubular cell injury. ROC analysis showed good discriminatory ability for BUN (area under the curve [AUC] = 0.827) and uNAG (AUC = 0.813). Therefore, uNAG may have potential utility as a non-invasive biomarker of early renal tubular injury in populations chronically exposed to Cd/Pb.

## Introduction

Chronic kidney disease (CKD) presents a growing challenge to public health systems globally, with substantial impacts on quality of life, healthcare costs, and premature mortality [1,2]. More than 850 million people worldwide are affected by kidney disease, particularly those with diabetes, hypertension, or long-term exposure to environmental pollutants [2]. In Thailand, the rising prevalence of type 2 diabetes (T2D) and hypertension over the past decade has significantly contributed to the burden of CKD, especially among older adults and vulnerable populations [3–5].

Plant-based diets have gained widespread attention for their role in preventing and managing non-communicable diseases (NCDs), including T2D, cardiovascular disease, and obesity [6–9]. Rich in fruits, vegetables, whole grains, legumes, nuts, and seeds, these diets provide antioxidants, fiber, and anti-inflammatory compounds that contribute to improved metabolic health and vascular function [10,11]. Because oxidative stress and systemic inflammation are common pathophysiological pathways shared between NCD and CKD [12], plant-based diets are increasingly being explored for their potential renoprotective effects [13–16].

However, in certain environmental settings, particularly in low- and middle-income countries, the benefits of plant-based diets may be undermined by the presence of foodborne contaminants [17,18]. Heavy metals such as cadmium (Cd) and lead (Pb), which are known nephrotoxicants [18,19], can accumulate in plant foods through polluted soil and water sources [15,17]. Chronic low-level exposure to these metals has been associated with tubular damage, proteinuria, and a higher risk of CKD, even among individuals without occupational exposure [14,20].

In such contexts, the early identification of kidney injury is crucial. While estimated glomerular filtration rate (eGFR) and serum creatinine are common measures [21], they may not capture early-stage renal damage [22]. Urinary N-acetyl-β-D-glucosaminidase (uNAG), released during proximal tubular injury, has been proposed as a sensitive biomarker for detecting subclinical renal impairment [22,23].

In southern Thailand, the Pak Phun subdistrict of Nakhon Si Thammarat Province has been identified as an area contaminated with heavy metals, especially Pb [24,25]. Plant food consumption is particularly common in this area [6,26], but the dual role of plant foods, as both protective and potential sources of nephrotoxic exposure, remains poorly understood.

This study investigated the relationships between plant food consumption, exposure to environmental Cd and Pb, and an early warning sign of kidney injury, assessed with urinary NAG. Study subjects were adult residents of a community in southern Thailand with known Pb contamination. The findings of this study may inform evidence-based dietary recommendations, guide environmental risk management, and support early CKD detection strategies for vulnerable populations.

## Materials and methods

### Study design and setting

This cross-sectional study employed purposive sampling to recruit individuals aged 50 years or older who had undergone chronic disease screening at a local health center in a community with documented environmental heavy metal contamination in southern Thailand between October and December 2024. Eligible participants were residents of the study area who had previously participated in chronic disease screening or were apparently healthy and could communicate effectively. The exclusion criteria were unwillingness or inability to provide complete information, unavailability during data collection, pregnancy or breastfeeding, and a medical history of advanced chronic illness.

The required sample size was calculated using a single-population proportion formula for cross-sectional studies. With an expected prevalence of screening uptake of 50%, a 95% confidence level (Z = 1.96), and a 10% margin of error (d = 0.10), the minimum required sample size was 96 participants.


$$\begin{array}{c} n=\frac{z^{\text{2}}*P(\text{1}-P)}{d^{\text{2}}}=\;\frac{{\text{1}.\text{9}\text{6}}^{\text{2}}*\text{0}.\text{5}(\text{1}-\text{0}.\text{5})}{{\text{0}.\text{1}\text{0}}^{\text{2}}}=\text{9}\text{6}.\text{0}\text{4} \end{array}$$ (1)

### Body composition measurement

Anthropometric measurements, including height and weight, were obtained using a height and weight scale machine (NAGATA MODEL BW-1122H, Tainan, Taiwan) and used to calculate body mass index (BMI, kg/m²). Waist circumference was measured using a measuring tape (Seca, Hamburg, Germany) following standard protocols.

Body composition parameters, including body fat percentage (%), muscle mass (kg), bone mass (kg), and basal metabolic rate (BMR), were assessed using a bioelectrical impedance analysis device (Tanita SC-330, Tanita Health Equipment, Kowloon, Hong Kong). The BMR was estimated using the device’s proprietary algorithms, which are based on demographic and body composition parameters.

### Dietary evaluation and estimation of plant-based food intake

Dietary intake was assessed using a validated Food Frequency Questionnaire (FFQ), which captured consumption frequency and typical portion size over a defined period. A Plant Food Score (PFS) was calculated based on the intake of five core categories of plant-based foods: vegetables, fruits, legumes, nuts/seeds, and cereals. The scoring method was adapted from a previously published study conducted in southern Thailand, which demonstrated the association between plant food consumption and glycemic control in patients with type 2 diabetes. Dietary intake levels for each plant food category (vegetables, fruits, legumes, nuts/seeds, and cereals) were first adjusted for body weight (g/kg). These values were then categorized into low, medium, and high groups based on quartile distribution. Quartile cut-off points (Q1 and Q3) were calculated separately for each category due to differences in intake distributions. Values <Q1 were defined as low intake, Q1–Q3 as medium intake, and >Q3 as high intake. Each category was subsequently assigned a score of 0, 1, and 2, respectively, and summed to obtain the total Plant Food Score (PFS), with higher scores indicating greater consumption of plant-based foods [6]. The actual PFS cut-off values used to classify consumption levels as low, medium, and high are provided in S1 Table.

### Blood and urine sampling

Blood and morning urine samples were collected from each participant for the analysis of renal biomarkers and heavy metal concentrations in the samples. Specifically, cadmium (Cd) and lead (Pb) levels were measured in whole blood samples, and N-acetyl-β-D-glucosaminidase (uNAG) was analyzed as a marker of renal tubular function.

To ensure accuracy in heavy metal analysis, standardized collection protocols were followed. Approximately 3 mL of venous blood was drawn using sterile techniques and transferred to ethylene diamine tetra-acetic acid (EDTA) tubes. Urine samples were collected in metal-free polypropylene containers to minimize the risk of contamination. All samples were promptly stored at –20°C until laboratory analysis to maintain sample integrity.

Internal quality control procedures were implemented during the analytical process. Duplicate and blank samples were used to monitor the consistency and potential contamination. Certified reference materials (CRMs) were used to validate the accuracy of blood Cd and Pb measurements. All procedures were conducted in accordance with the Good Laboratory Practice (GLP) standards to ensure data reliability and reproducibility.

### Lead and cadmium analysis

Blood concentrations of Cd and Pb were measured using graphite furnace atomic absorption spectrometry (GFAAS) with a GBC System 5000, 902 atomic absorption spectrophotometer, and a PAL2000 autosampler (GBC Scientific Equipment, Hampshire, IL, USA). The analytical method was based on the protocol described by Trzcinka-Ochockh et al. (2016) [27]. The limits of detection were 3 µg/dL for Pb and 0.1 µg/L for Cd. Calibration was performed using multi-element standard. Analytical precision and accuracy were verified using certified reference materials, including urine and whole blood controls, at three concentration levels (Lyphochek, Bio-Rad, Hercules, CA, USA). The recovery rates for Cd and Pb ranged between 90% and 110%, indicating acceptable analytical accuracy of the method. To maintain analytical reliability, external quality assessments for Pb and Cd were conducted every three years. All materials used in sample preparation, including tubes, bottles, and pipettes, were acid-washed and rinsed with deionized water before use.

### Cadmium and lead exposure profiling

To evaluate the combined effects of cadmium (Cd) and lead (Pb) exposure on renal function, participants were classified into three exposure profiles based on their blood Cd and Pb levels. The median values observed in this study were 0.30 µg/L for Cd and 3.45 µg/dL for Pb, which were used as the cutoff points. Participants in Exposure Profile 1 (low exposure) had blood Cd and Pb levels of < 0.30 µg/L and ≤ 3.45 µg/dL, respectively. In Profile 2 (moderate exposure) included individuals in whom the indicated levels of the analyzed elements were exceeded in only one case, either blood Cd ≥ 0.30 µg/L or blood Pb > 3.45 µg/dL. In Profile 3 (high exposure), both parameters exceeded the indicated levels (blood Cd ≥ 0.30 µg/L and blood Pb > 3.45 µg/dL). This classification enabled further analysis of the potential dose–response relationships between metal exposure and renal health outcomes, particularly focusing on N-acetyl-β-D-glucosaminidase (uNAG), blood urea nitrogen (BUN), and estimated glomerular filtration rate (eGFR).

### Measurement of urinary N-Acetyl-β-D-Glucosaminidase (uNAG)

N-acetyl-β-D-glucosaminidase (uNAG), an early biomarker of renal tubular injury, was measured using the β-N-acetylglucosaminidase Assay Kit (Catalog. No. CS0780, Sigma-Aldrich, USA). The assay is based on the enzymatic hydrolysis of 4-Nitrophenyl N-acetyl-β-D-glucosaminide (NP-GlcNAc), which releases p-nitrophenol, a product that can be measured at 405 nm after ionization under alkaline conditions. Briefly, 100 µL of urine sample was mixed with 100 µL of substrate solution in a 96-well microplate and incubated at 37 °C for 10 minutes. The reaction was stopped by adding 200 µL of stop solution (sodium carbonate), and the absorbance was measured immediately at 405 nm using a microplate reader (BioTek, USA). Each sample was run in triplicate. Enzyme activity was calculated based on the standard curve of p-nitrophenol and expressed in units per liter (U/L), where one unit corresponds to the hydrolysis of 1 µmol of substrate per minute at pH 4.7 and 37 °C. Urinary creatinine concentration was determined using the Jaffe’s alkaline picrate method [28] and used to normalize uNAG levels expressed as U/mmol creatinine.

### Ethical considerations

This study was approved by the Human Research Ethics Committee of Walailak University (approval number WUEC-24-275-01). Written informed consent was obtained from all participants prior to data collection. This study was conducted in accordance with the ethical principles of the Declaration of Helsinki and the relevant institutional guidelines.

### Statistical analysis

Statistical analyses were performed using SPSS version 28 (IBM Inc., NY, USA) and GraphPad Prism version 10 software (GraphPad, CA, USA). Continuous variables are presented as mean ± standard deviation (SD), while data in figures are presented as mean ± standard error of the mean (SEM). For comparisons between two groups, the Mann-Whitney U test was used; for three or more groups, one-way ANOVA or the Kruskal-Wallis test was applied, followed by Bonferroni post hoc analysis as appropriate. Categorical variables were compared using Pearson’s chi-square tests.

Multiple linear regression was conducted to assess the association between the PFS and renal biomarkers (BUN, eGFR, and uNAG). Multivariable logistic regression was performed to assess the odds of elevated uNAG (≥ 0.77 U/mmolCr) in relation to plant food groups, adjusting for sex, age, and smoking status.

Tubular biomarkers, including uNAG, were analyzed alongside conventional markers (eGFR and BUN) to assess their potential utility for early detection of renal impairment in populations exposed to heavy metals. The discriminatory ability of BUN, eGFR, and uNAG for CKD detection was assessed using receiver operating characteristic (ROC) curve analysis, with area under the curve (AUC) and 95% confidence intervals. A p-value < 0.05 was considered statistically significant for all analyses.

## Results

### Participant characteristics by exposure profile

A total of 96 participants were included in the study, with 34 in Profile 1, 34 in Profile 2, and 28 in Profile 3. Overall, 81.2% were women, with a mean age of 61.73 ± 10.06 years. A significant difference in smoking status was observed across the exposure profiles (p = 0.021), with the highest proportion of smokers in Profile 3 (25.0%). Although the proportion of smokers was highest in Profile 3 (25%), 21 participants (75%) in this group were non-smokers. This finding suggests that non-smoking-related sources, particularly environmental contamination and occupation-related exposure (e.g., fishing-related activities in the study area), may substantially contribute to Cd and Pb exposure in this population [29]. Anthropometric indicators, including BMI, waist circumference, and body fat percentage, showed a trend of increase across exposure groups; however, these differences were not statistically significant.

Regarding renal function, blood urea nitrogen (BUN) increased significantly with higher exposure, from 12.64 ± 3.05 mg/dL in Profile 1 to 15.79 ± 5.72 mg/dL in Profile 3 (p = 0.016). Although not statistically significant, the eGFR and uNAG levels demonstrated directional changes consistent with early renal impairment. Metabolic parameters, such as fasting blood glucose and systolic blood pressure, were higher in participants with greater heavy metal exposure (profile 3) but did not reach statistical significance.

The prevalence of diabetes and hypertension differed significantly across the exposure profiles (p = 0.016 and p = 0.024, respectively). Diabetes prevalence was highest in Profile 1, whereas hypertension was highest in Profile 3. In contrast, the prevalence of chronic kidney disease did not differ significantly (p = 0.073). As expected, the mean blood levels of Cd and Pb increased progressively across the profiles, supporting the validity of exposure classification. Participants in Profile 3 exhibited the highest concentrations of both metals (7.60 ± 4.67 μg/dL for Pb and 1.16 ± 0.67 μg/L for Cd; Table 1).

**Table 1:** Characteristics of participants grouped by their Cd and Pb exposure profiles.

Characteristic	All Participants (n = 96)	Cadmium and Lead Exposure			p-value
		Profile 1 (n = 34)	Profile 2 (n = 34)	Profile 3 (n = 28)	
**Sex**, n (%)					
Male	18 (18.8%)	3 (8.8%)	8 (23.5%)	7 (25.0%)	0.180
Female	78 (81.2%)	31 (91.2%)	26 (76.5%)	21 (75.0%)	
**Age** (years), mean ± SD	61.73 ± 10.06	61.68 ± 11.01	62.32 ± 10.06	61.07 ± 9.11	0.889
**Smoking**, n (%)					
No	85 (88.5%)	33 (97.1%)	31 (91.2%)	21 (75.0%)	0.021*
Yes	11 (11.5%)	1 (2.9%)	3 (8.8%)	7 (25.0%)	
**Anthropometric data**					
BMI (kg/m²)	25.48 ± 5.11	24.97 ± 4.26	25.32 ± 4.23	26.28 ± 6.84	0.598
Waist (cm)	91.22 ± 11.34	90.21 ± 10.04	90.85 ± 9.92	92.89 ± 14.30	0.637
Fat (%)	33.61 ± 9.57	35.04 ± 6.17	32.01 ± 10.27	33.83 ± 11.87	0.428
Muscle mass (Kg)	38.49 ± 6.83	37.48 ± 6.92	39.30 ± 6.46	38.73 ± 7.23	0.539
Bone mass (Kg)	2.29 ± 0.40	2.25 ± 0.41	2.33 ± 0.33	2.30 ± 0.47	0.729
BMR (kcal/day)	1288.99 ± 953.18	1168.82 ± 217.78	1218.00 ± 199.90	1189.93 ± 220.02	0.485
**Renal function**					
BUN (mg/dL)	13.78 ± 4.53	12.64 ± 3.05	13.27 ± 4.25	15.79 ± 5.72	0.016*
Urine creatinine (mg/dL)	91.78 ± 56.19	91.44 ± 52.38	93.01 ± 54.14	90.70 ± 64.57	0.986
eGFR (mL/min/1.73 m²)	79.85 ± 19.41	83.53 ± 13.77	79.23 ± 20.44	76.15 ± 23.50	0.324
uNAG (U/mmolCr)	1.18 ± 1.70	0.62 ± 0.37	1.56 ± 2.17	1.40 ± 1.89	0.052
**Metabolic parameters**					
FBS (mg/dL)	133.54 ± 66.91	114.97 ± 33.67	142.21 ± 87.90	145.57 ± 65.16	0.129
SBP (mmHg)	138.71 ± 17.40	137.41 ± 17.28	135.65 ± 15.71	144.00 ± 18.88	0.148
DBP (mmHg)	83.39 ± 9.71	84.53 ± 9.75	80.21 ± 9.08	85.86 ± 9.71	0.050
**Heavy metal exposure**					
Blood Pb (µg/dL)	5.39 ± 5.37	2.28 ± 0.36	6.69 ± 7.01	7.60 ± 4.67	–
Blood Cd (µg/L)	0.53 ± 0.68	0.09 ± 0.10	0.46 ± 0.65	1.16 ± 0.67	–
**Chronic Condition**					
Diabetes	47 (49.0%)	23 (67.6%)	15 (44.1%)	9 (32.1%)	0.016*
Hypertension	48 (50.0%)	15 (44.1%)	13 (38.2%)	20 (71.4%)	0.024*
Chronic Kidney Disease	16 (16.7%)	2 (5.9%)	9 (26.5%)	5 (17.9%)	0.073

BMI, body mass index; BMR, basal metabolic rate; BUN, blood urea nitrogen; eGFR, estimated glomerular filtration rate; uNAG, urinary N-acetyl-β-D-glucosaminidase; FBS, fasting blood sugar; SBP, systolic blood pressure; DBP, diastolic blood pressure; Pb, lead; Cd, cadmium; CKD, chronic kidney disease. CKD was defined as eGFR < 60 mL/min/1.73 m². Data are mean ± SD or n (%). Group differences were assessed using the Kruskal-Wallis or chi-square test. p < 0.05 was considered significant.

### Plant food consumption and kidney health across exposure profiles

**Fig 1:**
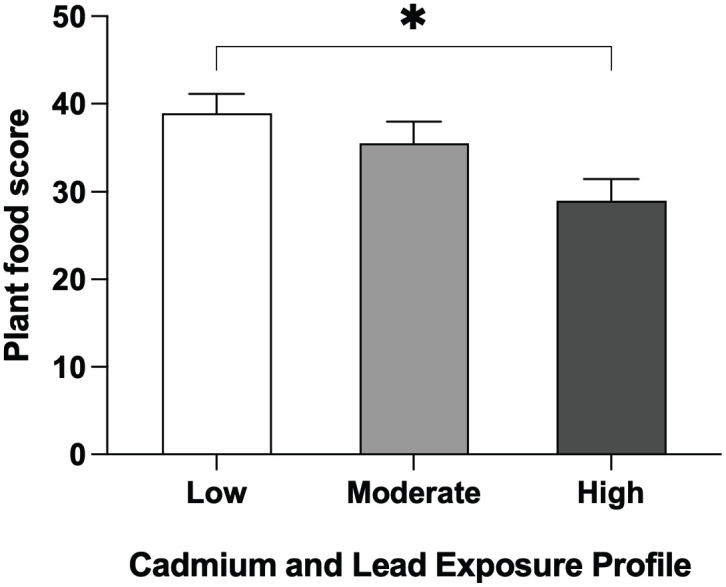
Plant food consumption across Cd and Pb exposure profiles. Participants with low Cd/Pb exposure had significantly higher plant food scores than those with high exposure (*p < 0.05). Data are presented as mean ± SEM.

**Fig 2:**
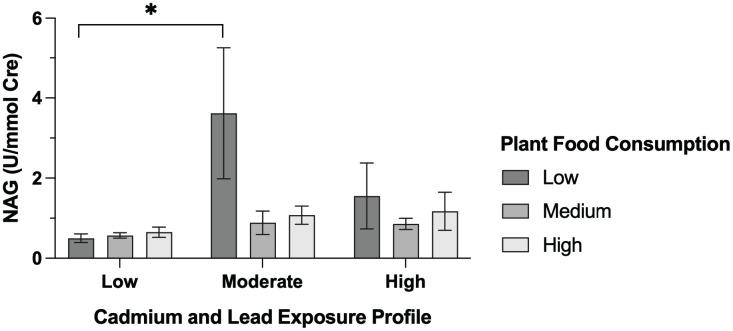
uNAG levels by plant food consumption across heavy-metal exposure profiles. In the moderate exposure group, participants with low plant food intake had significantly higher uNAG levels than those with medium or high intake of plant foods (*p < 0.05). Data are presented as mean ± SEM.

PFS differed significantly across Cd and Pb exposure profiles, with the highest scores observed in the low exposure group and the lowest in the high exposure group (Fig 1). When stratified by intake level, participants in the moderate exposure group with low plant food consumption had notably higher uNAG levels than those with medium or high intake (Fig 2), suggesting a potential mitigating effect of plant food against tubular injury in this group. In Multiple linear regression analysis, total plant food consumption scores were inversely associated with uNAG (β = –0.297, p = 0.010), but not with BUN, urine creatinine, or eGFR (Table 2).

**Table 2:** Associations between plant food consumption scores and kidney function indicators.

kidney function indicators	B	SE	β	p-value
BUN (mg/dL)	–0.239	0.368	–0.077	0.518
Urine creatinine (mg/dL)	–0.028	0.026	–0.113	0.274
eGFR (mL/min/1.73 m²)	–0.169	0.090	–0.234	0.064
uNAG (U/mmolCr)	–2.448	0.933	–0.297	0.010*

Multiple linear regression was used. B, unstandardized coefficient; SE, standard error; β, standardized coefficient; BUN, blood urea nitrogen; eGFR, estimated glomerular filtration rate; uNAG, urinary N-acetyl-β-D-glucosaminidase (U/mmolCr). p < 0.05 was considered significant.

### Discriminatory ability of kidney function indicators for detecting chronic kidney disease

**Fig 3:**
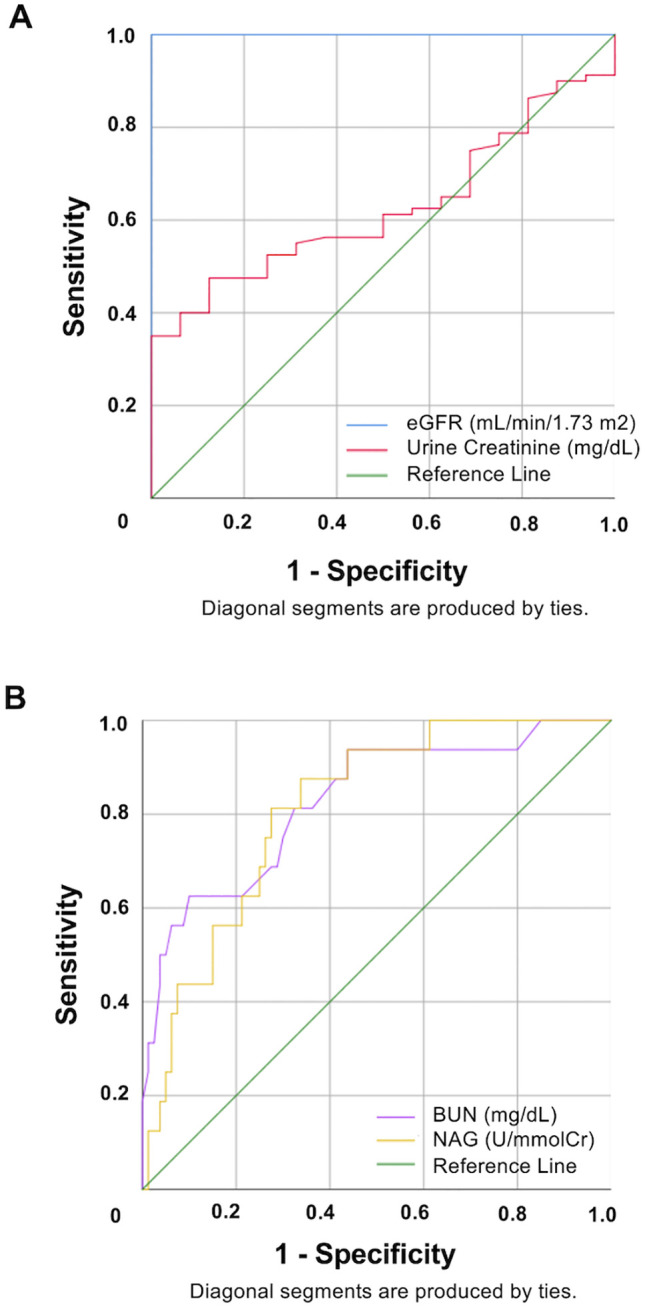
Receiver operating characteristic (ROC) curves of kidney function indicators for detecting chronic kidney disease (CKD). (A) ROC curves for eGFR and urine creatinine. Urine creatinine showed limited discriminatory ability (AUC = 0.626). (B) ROC curves for BUN and uNAG. Both BUN (AUC = 0.827) and uNAG (AUC = 0.813) showed good discriminatory ability. The optimal cutoff value for uNAG was 0.77 U/mmolCr, with 88% sensitivity and 66% specificity.

ROC analysis was performed (Fig 3, Table 3). eGFR showed perfect discrimination (AUC = 1.000, p < 0.001), which was expected because CKD status was defined using an eGFR threshold. In contrast, urine creatinine showed limited discriminatory ability. Both BUN (AUC = 0.827) and uNAG (AUC = 0.813) demonstrated good discriminatory ability (p < 0.001). The optimal cutoff value for uNAG was 0.77 U/mmolCr, with 88% sensitivity and 66% specificity, supporting its potential utility as a non-invasive biomarker of early renal tubular injury in environmentally at-risk populations.

**Table 3:** Discriminatory ability of kidney function indicators for detecting chronic kidney disease using ROC curve analysis.

Kidney Function Indicators	AUC ROC	SE	95%CI	Cutoff	Sensitivity	Specificity	p-value
eGFR (mL/min/1.73 m²)	1.000	0.000	1.00-1.00	–	–	–	<0.001**
Urine creatinine (mg/dL)	0.626	0.060	0.51-0.74	–	–	–	0.112
BUN (mg/dL)	0.827	0.060	0.71-0.94	–	–	–	<0.001**
uNAG (U/mmolCr)	0.813	0.050	0.71-0.91	0.77	0.88	0.66	<0.001**

AUC, area under the curve; SE, standard error; CI, confidence interval; eGFR, estimated glomerular filtration rate; BUN, blood urea nitrogen; uNAG, urinary N-acetyl-β-D-glucosaminidase (U/mmolCr). CKD was defined as eGFR < 60 mL/min/1.73 m². Cutoff values were identified using the Youden index. AUC > 0.5 indicates discriminatory ability. p ≤ 0.05 was considered significant.

### Association between plant food consumption and renal tubular damage

No significant associations were observed between the total PFS and the intake of vegetables, fruits, legumes, or cereals. However, participants with a high intake of nuts and seeds had a significantly lower likelihood of elevated uNAG compared with those with a low intake (adjusted OR = 0.185, 95% CI: 0.040–0.860, p = 0.032). These results may suggest a potential association between high consumption of nuts and seeds and the maintenance of tubular cell integrity (Table 4).

**Table 4:** Multivariable logistic regression analysis of the association between plant food consumption and elevated uNAG levels.

PFS	uNAG < 0.77(U/mmolCr) n = 55 (%)	uNAG ≥ 0.77 (U/mmolCr) n = 41 (%)	Adjusted OR	95%CI	p-value
Total PFS					
Low	12 (21.8%)	10 (24.4%)	Ref		
Medium	29 (52.7%)	16 (39.0%)	0.670	0.23-1.93	0.458
High	14 (25.5%)	15 (36.6%)	1.095	0.35-3.46	0.878
Vegetables					
Low	15 (27.3%)	11 (26.8%)	Ref		
Medium	33 (60.0%)	17 (41.5%)	1.061	0.24-4.63	0.937
High	7 (12.7%)	13 (31.7%)	4.606	0.76-27.80	0.096
Fruits					
Low	16 (29.1%)	10 (24.4%)	Ref		
Medium	23 (41.8%)	21 (51.2%)	1.624	0.36-7.41	0.531
High	16 (29.1%)	10 (24.4%)	0.977	0.17-5.64	0.979
Legumes					
Low	16 (29.1%)	9 (22.0%)	Ref		
Medium	26 (47.3%)	25 (61.0%)	1.90	0.59-6.01	0.281
High	13 (23.6%)	7 (17.1%)	0.724	0.18-2.87	0.646
Nuts/seeds					
Low	16 (29.1%)	18 (43.9%)	Ref		
Medium	21 (38.2%)	16 (39.0%)	0.366	0.10–1.40	0.141
High	18 (32.7%)	7 (17.1%)	0.185	0.04-0.86	0.032*
Cereals					
Low	15 (27.3%)	13 (31.7%)	Ref		
Medium	25 (45.5%)	15 (36.6%)	0.833	0.19-3.74	0.812
High	15 (27.3%)	13 (31.7%)	1.365	0.25-7.58	0.722

uNAG, urinary N-acetyl-β-D-glucosaminidase (U/mmolCr); PFS, plant food score (0 = low, 1 = medium, 2 = high intake); OR, odds ratio; CI, confidence interval; Ref, lowest intake group. Elevated uNAG was defined as ≥0.77 U/mmolCr. Logistic regression was adjusted for sex, age, and smoking status. p <0.05 was considered significant.

## Discussion

The present study examined potential associations between consumption of plant foods, kidney function indicators (BUN, eGFR) and a reliable marker of kidney tubular cell injury (uNAG) in middle-aged adults who were exposed to environmental Cd and Pb. Higher PFS were associated with lower uNAG levels, but they were not associated with BUN, urinary creatinine, or eGFR (Table 2). Among specific food groups, a higher intake of nuts and seeds was associated with lower uNAG levels (Table 4). These findings may suggest a potential association between kidney tubule integrity and consumption of nuts and seeds. Participants in the higher exposure profiles tended to have lower eGFR and higher uNAG levels than those in the low-exposure profile (Table 1). These results were consistent with numerous literature reports on effects of Cd and Pb exposure on kidney damage and a falling eGFR even exposure levels were low as experienced by participants in the present study [22,30]

The insignificant associations between PFS and conventional renal function indicators notably BUN, urine creatinine, and eGFR may reflect sensitivity differences. The estimated glomerular filtration rates (eGFR) often remain within a normal range until substantial nephron loss has occurred, limiting their utility in early detection of renal functional impairment, especially in individuals with chronic diseases such as diabetes and hypertension [3,4,12]. Apparently, uNAG was a more sensitive marker of proximal tubular injury, and may better reflect early renal impairment driven by oxidative stress and inflammation [31]. These are the mechanisms through which plant chemicals such as flavonoids, polyphenols, and unsaturated fatty acids may help protect the kidneys [10–12,32]. Chronic exposure to Cd and Pb may further enhance tubular vulnerability and obscure associations with filtration markers, even at low-to-moderate exposure levels, which has been linked to metabolic and renal dysfunction in general and high-risk populations [15,20,33]. In support of this notion, a recent longitudinal cohort study among Mexican Americans reported that higher urinary levels of several toxic metals and toxic metal mixtures were associated with faster worsening of glycemic traits and earlier progression toward developing diabetes [34]. These findings underscore the broader metabolic consequences of chronic environmental metal exposure.

Generally, plant foods consumption is regarded as protective against chronic disease; however, not all plant foods contribute equally to renal outcomes. Herein, nuts and seeds showed a strong inverse association with uNAG. This aligns with previous reports demonstrating beneficial effects of these foods particularly on metabolic and renal health [6,11]. Their protective effects could be attributed to specific chemicals that include unsaturated fatty acids, vitamin E, and polyphenols, which help mitigate oxidative stress damage and inflammation, which are key mechanisms in CKD pathogenesis [10,11]. Nuts and seeds are particularly rich in magnesium, arginine, and unsaturated fatty acids [35]. These chemicals are known to enhance nitric oxide–mediated endothelial function, improve renal microvascular integrity, and support peritubular perfusion, thereby reducing tubular hypoxia [36,37]. In addition, they may improve lipid profiles and insulin sensitivity and reduce oxidative stress and inflammation, which are key contributors to tubulointerstitial damage and CKD progression [38,39]. Given the high prevalence of chronic metabolic conditions, such as diabetes (49.0%) and hypertension (50.0%), among participants, these results underscore the importance of not only promoting plant-based diets but also emphasizing the quality and specific composition of plant food choices. Nuts and seeds, in particular, have a low glycemic index and anti-inflammatory properties, making them especially beneficial for individuals at risk of renal impairment. These findings suggest that dietary interventions for kidney health should move beyond generalized recommendations for plant-based diets. For instance, specific plant food groups with potential nephroprotective effects should be highlighted.

Our findings reaffirm associations between signs of nephrotoxicity and chronic environmental exposure to Cd and Pb. The high Cd/Pb-exposure group had higher mean uNAG and lower mean eGFR, compared to the low-exposure group (Table 1). These results were in line with earlier studies that linked low-to-moderate Cd and Pb exposure to renal impairment [33], while pre-existing metabolic disorders may impair kidney function in vulnerable populations. In the context of Pak Phun, a region with known Pb contamination [6,24], these data highlight a hidden health risk of residents who may appear healthy or manage their chronic diseases through dietary changes, while unknowingly remaining at risk due to environmental or dietary exposure.

The findings also support the utility of uNAG as an early warning sign of injury to kidney tubular cells [22,40]. With an AUC of 0.813 (Table 3, Fig 3), uNAG showed good discriminatory ability, in detecting subclinical kidney damage before changes in eGFR occur [31]. Its noninvasive nature makes it a promising screening tool, particularly in high-risk areas such as Pak Phun. However, the cross-sectional design limits causal interpretation, and dietary heavy metal content was not directly measured in this study. Potential confounding from unmeasured variables, such as disease duration, medication use, or occupational exposure, may also exist. Smoking is a known source of Cd and Pb that could potentially confound the results. Therefore, we acknowledge that a lack of quantitative data on smoking intensity (e.g., number of cigarettes per day) was another limitation. To address this issue, smoking status was included as a covariate in the multivariable regression models (Table 4). Future studies should be focused on longitudinal designs that incorporate dietary metal quantification, environmental sampling, and serial biomarker testing to clarify the causal pathways.

## Conclusions

A higher intake of nuts and seeds was associated with a lower level of kidney tubular cell injury, reflected by uNAG levels. This result may suggest a potential health benefit and nutritional values of nuts and seeds. Concerningly, however, such health benefits associated with plant food consumption may be diminished in those residing in areas, endemically contaminated with Cd and Pb. Therefore, health promotion strategies in vulnerable communities should combine access to beneficial plant foods with efforts to minimize environmental Cd and Pb exposure. Another notable finding was the potential utility of uNAG in detecting subclinical tubular injury.

## Supporting information

S1 Dataset Minimal dataset used to replicate the study findings. (XLSX)

S1 Table Cut-off values for plant food intake categories. (DOCX)
