# Supplementary material for: Role for plant foods in kidney health among middle-aged individuals environmentally exposed to cadmium and lead
Source: PLoS One. 2026 May 29;21(5):e0350506. doi: 10.1371/journal.pone.0350506 (PMC13221064; doi:10.1371/journal.pone.0350506)
Supplement: S1 Table — (DOCX) [file pone.0350506.s002.docx]

**S2 Table** Cut-off values of Plant Food Scores to categorize consumption levels as low, medium, and high.

| **Plant Food Score** | **Low** | **Medium** | **High** |
| --- | --- | --- | --- |
| Vegetables | <7 | 7–14 | >14 |
| Fruits | <4 | 4–10 | >10 |
| Legumes | <5 | 5–12 | >12 |
| Nuts/seeds | 0 | 1–5 | >5 |
| Cereals | <4 | 4–8 | >8 |
| Total PFS | <27 | 27–43 | >43 |
